# Intuitive Terrain Reconstruction Using Height Observation-Based Ground Segmentation and 3D Object Boundary Estimation

**DOI:** 10.3390/s121217186

**Published:** 2012-12-12

**Authors:** Wei Song, Kyungeun Cho, Kyhyun Um, Chee Sun Won, Sungdae Sim

**Affiliations:** 1 Department of Multimedia Engineering, Dongguk University-Seoul, 26 Pildong 3 Ga, Jung-gu, Seoul 100-715, Korea; E-Mails: songwei@dongguk.edu (W.S.); khum@dongguk.edu (K.U.); 2 Division of Electronics and Electrical Engineering, Dongguk University-Seoul, 26 Pildong 3 Ga, Jung-gu, Seoul 100-715, Korea; E-Mail: cswon@dongguk.edu; 3 Agency for Defense Development, Bugyuseong daero 488 beon gi, Yoseong, Daejeon 305-152, Korea; E-Mail: sdsim@add.re.kr

**Keywords:** terrain reconstruction, 3D ground segmentation, 3D boundary estimation, height histogram, Gibbs-Markov Random Field

## Abstract

Mobile robot operators must make rapid decisions based on information about the robot’s surrounding environment. This means that terrain modeling and photorealistic visualization are required for the remote operation of mobile robots. We have produced a voxel map and textured mesh from the 2D and 3D datasets collected by a robot’s array of sensors, but some upper parts of objects are beyond the sensors’ measurements and these parts are missing in the terrain reconstruction result. This result is an incomplete terrain model. To solve this problem, we present a new ground segmentation method to detect non-ground data in the reconstructed voxel map. Our method uses height histograms to estimate the ground height range, and a Gibbs-Markov random field model to refine the segmentation results. To reconstruct a complete terrain model of the 3D environment, we develop a 3D boundary estimation method for non-ground objects. We apply a boundary detection technique to the 2D image, before estimating and refining the actual height values of the non-ground vertices in the reconstructed textured mesh. Our proposed methods were tested in an outdoor environment in which trees and buildings were not completely sensed. Our results show that the time required for ground segmentation is faster than that for data sensing, which is necessary for a real-time approach. In addition, those parts of objects that were not sensed are accurately recovered to retrieve their real-world appearances.

## 1. Introduction

Remote operation of mobile robots is widely used in planetary exploration, search and rescue, surveillance, defense, and other robotic applications [1]. An operator controls the mobile robot through a remote control system (RCS), which provides an immersive virtual environment to enable an understanding of terrain information [2–4]. The operator controls the mobile robot by navigating and interacting with real environments without collisions or encountering other dangers [5]. In situations where the operator must quickly decide on the motion and path of the robot, rapid feedback of the real environment is vital for effective control, so real-time terrain modeling and photorealistic visualization systems have been developed [6].

**Figure 1.**
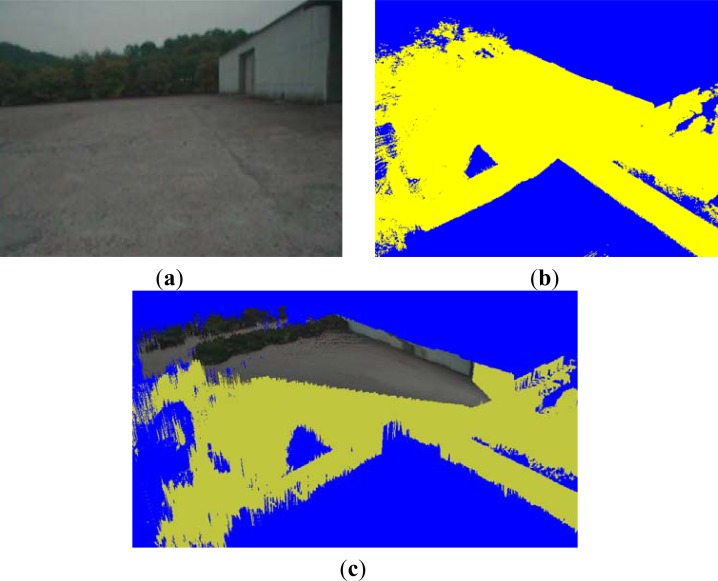
Terrain models. (**a**) Captured 2D image. (**b**) Voxel map. (**c**) Textured mesh.

Conventional real-time visualization systems mostly apply a 2D image, a voxel map or a texture map to represent a terrain model. A 2D image is captured by the mobile robot’s camera. For example, the 2D image in Figure 1(a) is captured by the camera on the front of a robot. A voxel map, as shown at a quarter viewpoint in Figure 1(b), is generated by integrating the sensed 3D point clouds into regular grids. From the voxel map, a terrain mesh, as shown at quarter viewpoint in Figure 1(c), is generated by integrating the top points in the *x–z* cells into a regular triangular mesh. By mapping the texture in Figure 1(a) onto the mesh, a textured mesh is obtained [7]. The yellow vertices in Figure 1(c) denote the regions that are not projected from the 2D image. A terrain model consisting of geometrical shapes and realistic textures enables a photorealistic visualization approach for the terrain reconstruction and a remote operation of mobile robots.

In large-scale environments, a level-of-detail (LOD) method is used to render the near-field regions of the terrain model. In far-field regions, billboard rendering methods [8], which represent a texture in front of the terrain model for real-time visualization, are applied. However, when processing a terrain model, the upper regions of objects are often outside the measurement range of the 3D sensor. These “unsensed” parts of large objects exist in the reconstructed terrain model. In Figure 1(c), we can see that the top parts of the buildings and trees are missing in the terrain reconstruction result. We need to recover the missing parts of tall objects. The objective of our study is to reconstruct a complete terrain model with object detection and 3D boundary estimation of non-ground objects.

**Figure 2.**
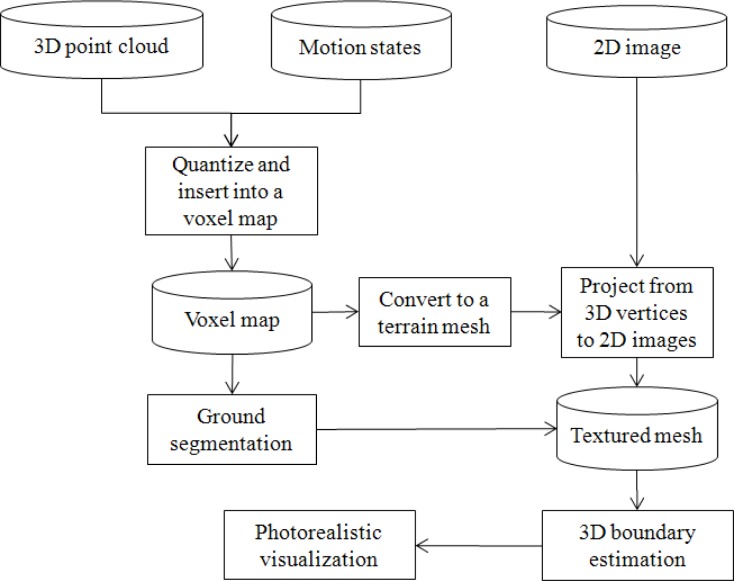
Framework for terrain modeling and photorealistic visualization using ground segmentation and 3D boundary estimation.

In this paper, we aim to constitute a real-time, large-scale terrain modeling system for photorealistic visualization, including our new ground segmentation method and 3D boundary estimation algorithm. The framework of the proposed system is shown in Figure 2.

The system includes three principal steps. Firstly, data from the integrated sensors are used to generate a voxel map and a textured mesh as terrain models. The multiple sensors mounted on mobile robots collect terrain information in the form of 3D point clouds, 2D images, GPS, and rotation states. Based on the rotation and position data, the received 3D point clouds are transformed to absolute positions which are quantized into regular grids and registered into a voxel map and a textured mesh by projection from vertices to 2D images.

Next, we develop a ground segmentation method to classify ground surface and non-ground objects in the voxel map. We apply a height histogram method, based on the spatial distribution of the ground and objects, to segment ground data in the voxel map. Because the voxels in the terrain model are highly affected by their neighbors, we apply a Gibbs-Markov random field (GMRF) [9,10] to refine the segmentation result.

Finally, our 3D boundary detection algorithm is applied to recover unsensed parts of non-ground objects. The missing portions of objects are reconstructed by detecting the object boundary in the 2D image, then estimating the true height from the incomplete boundary in the textured mesh.

This paper is organized as follows: in Section 2, we survey related work on terrain modeling, ground segmentation, and photorealistic modeling methods. In Section 3, we explain a ground segmentation method for voxel maps. In Section 4, we describe our non-ground object boundary estimation method for complete terrain reconstruction. The performance of the proposed ground segmentation and photorealistic visualization methods are analyzed and evaluated in Section 5, and finally, in Section 6, we draw our conclusions.

## 2. Related Works

There are many approaches to terrain modeling motivated by techniques from large-scale voxel maps and textured meshes. For example, there are algorithms based on multiple-sensor integration, large-scale dataset registration, ground surface and non-ground objects reconstruction, and 3D point interpolation. In this section, we review the ground surface and non-ground objects reconstruction methods. In addition, we investigate non-ground the researches on objects segmentation and 3D point interpolation, in order to recover the unsensed parts of large objects in the reconstructed terrain model.

When we represent a robot’s surrounding terrain in a virtual environment, it is necessary to reconstruct a terrain model using an integrated dataset obtained from multiple sensors [11–15]. Conventionally, the voxel map [16] and textured mesh [2] have been applied for this terrain modeling.

Huber *et al.*[8] and Kelly *et al.*[12] described real-world representation methods using video-ranging modules. 3D textured voxel grids were used to describe the surrounding terrain in the near field, whereas a billboard texture in front of the robot was used to show scenes in the far field. When the virtual camera changed its position and rotation, the billboard could not match the rendering result from the 3D modeling. For different virtual camera motion, therefore, the far-field scene should be represented as it appears in the real world.

Noguera *et al.*[17] proposed a hybrid photorealistic visualization system with a 2D synthetic panorama generation method to provide on-line photorealistic visualization. The client system rendered the terrain close to the virtual camera using the LOD method. The far-field terrain was represented by a panorama, which was generated from a far-field terrain model rendered by a high-capability server system. However, it is difficult for these methods to estimate the extent of large objects when the 3D sensors cannot measure their heights. To solve this problem, we propose a non-ground object boundary estimation method to recover complete objects from the captured 2D image and the reconstructed terrain mesh.

A ground segmentation algorithm that classifies ground surface and non-ground objects in the reconstructed terrain models is necessary to recover unsensed parts of the non-ground objects. Conrad *et al.*[18] applied the scale-invariant feature transform (SIFT) algorithm [19] to establish a correspondence between pixels on stereo images. To cluster them into ground and non-ground classes, he used a modified Expectation Maximization algorithm. In his work, only the corresponding pixels were clustered. Ke *et al.*[20] improved Conrad’s method by constructing the contours of the image and judging whether a contour belongs to the ground plane. Because of the limited range and resolution of a stereo camera, only a small quantity of ground pixels could be obtained. A 3D sensor with highly accurate data collection is required to determine which areas are safe for a mobile robot.

Oniga *et al.*[21] utilized a random sample consensus (RANSAC) algorithm to detect a road surface and cluster obstacles based on the density of the sensed points, and Mufti *et al.*[22] presented a spatio-temporal RANSAC framework to detect planar surfaces. Based on the planar features of the ground, the detected area was then segmented. To improve the accuracy of the RANSAC plane, Lam *et al.*[23] proposed a least-squares fit plane with a Kalman filter to extract the road data from sequentially obtained 3D point clouds. Due to the computational cost of the RANSAC algorithm, it is difficult to apply this method in real-time ground segmentation approaches.

To segment ground data in the reconstructed terrain model, we need to calculate each voxel’s probability of being in the ground and non-ground configurations. An effective approach to object segmentation from 2D images and 3D point clouds is the Markov random field (MRF) algorithm [24–30].

Vernaza *et al.*[31] presented a prediction-based structured terrain classification method for the DARPA Grand Challenge. He used an MRF model to classify the pixels in 2D images into obstacles or ground regions. However, it is difficult to specify the probability density functions (PDFs) in MRFs. To solve this problem, the Hammersley-Clifford theorem proved an equivalence relationship between MRF and the Gibbs distribution [25]. Because the computation of GMRFs is too complicated for large-scale datasets, we need to remove redundant elements from the GMRF in order to reduce the computational cost of ground segmentation.

Song *et al.*[32] proposed a ground segmentation method in 2D images that combined the GMRF method with a flood-fill algorithm. By segmenting ground pixels in the 2D image, the method detects the ground vertices in the texture mesh by projecting from the ground pixels. Due to the computation requirements of image processing, it is not possible to apply ground segmentation for 2D images with real-time processing. In this paper, we propose a ground segmentation method for a 3D terrain mesh without image processing. The method applied a height histogram to estimate ground height range and a GMRF model to classify ground surface and non-ground objects in the voxel map. As it is different from the captured 2D images, the voxel map changes little with collection time. The processing duration of the method is less than that of the sensing duration of 3D point cloud. This way, the proposed method is able to realize real-time terrain reconstruction.

The recovery of unsensed regions plays a major role in obstacle avoidance. Some researchers have applied interpolation algorithms to fill empty holes and smooth terrain [33–36]. For example, when we estimate such unobserved data, Douillard *et al.*[37] interpolated grids in empty regions of elevation maps in order to propagate label estimates. This method represents a terrain map using a 3D textured voxel grid, and applies a point interpolation algorithm to fill any small holes. While successful in filling empty holes and smoothing terrain, this approach also encounters difficulties in estimating the height of large objects based on the 3D sensor measurements.

In hardware design research, Früh *et al.*[38] utilized a vertical laser scanner to measure large buildings and represent streetscapes in urban environments. When an object is located between the sensors and a building, some regions of the building are blocked by the object in the scanning results. These missing regions are filled by a planar or horizontal interpolation algorithm.

Point interpolation algorithms are used to fill small holes in the 3D grid. However, it is difficult for these methods to estimate the height of large objects, meaning that the actual shape of tall objects is often misrepresented. Point interpolation algorithms are also ineffective in representing porous objects, such as vegetation. To solve these problems, Song *et al.*[32] proposed a GMRF based height estimation algorithm by estimating object top pixel in 2D images for each sensed object pixel. He reconstructed the complete terrain from the captured 2D image and the reconstructed terrain mesh. The complex computation of GMRF causes a low speed of this method. We propose a boundary estimation method by a kernel-based boundary detection algorithm in 2D image. The top pixels of objects are easily detected by finding the boundary above the sensed pixels.

In this paper, we integrate a colored voxel map and a textured mesh to construct a photorealistic terrain model. For the ground segmentation in the 3D voxel map, we present a height histogram method with a GMRF model. Further, in contrast to interpolation methods, we explain a 3D boundary estimation method to recover unsensed regions in the textured mesh, especially for high or tall objects outside the sensors’ range of measurement.

## 3. Ground Segmentation in the Voxel Map

Before recovering the unsensed parts of non-ground objects, we require a ground segmentation algorithm that classifies ground and non-ground data from the reconstructed voxel map. We aim to autonomously segment ground surface in rough and slopy terrain environment and segment non-ground object with as few errors as possible. In this section, we apply a height histogram method and a GMRF model for this purpose. To initialize the variables in the GMRF model, such as height observation and configuration, in Section 3.1 we roughly segment the ground surface using a height histogram method based on the spatial distribution of ground surface and non-ground objects. Some errors will exist in this segmentation result. To remove these, we apply the GMRF model to refine the segmentation in Section 3.2. Then, from the non-ground voxel segmentation result, we will estimate the actual height value for the non-ground vertices. This procedure is described in Section 4.

### 3.1. Ground Height Range Estimation by Height Histogram

We usually segment the 3D points using the height of the robotic vehicle *h*_1_ as the standard. If the *y* coordinate of a 3D point is between −*h*_1_ − ∆ and −*h*_1_ + ∆, then we assume that this point is ground data. However, this method is not accurate in regions where the surface is sloped or rough, as the robot cannot move smoothly and the 3D sensor’s height value is unstable. In this section, we apply a height histogram method to estimate the ground height range in real time from the reconstructed voxel map.

**Figure 3.**
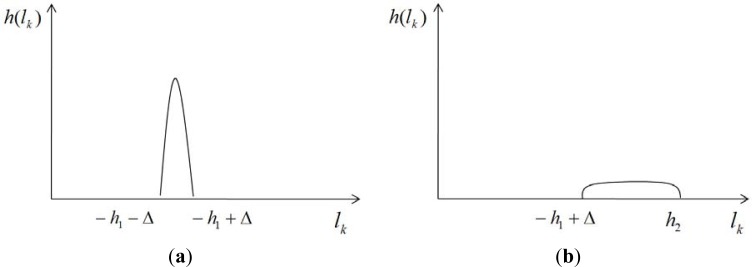
Histogram examples of height value distributions. (**a**) A height histogram for ground data. (**b**) A height histogram for non-ground data.

The height histogram is a graph representing the distribution of height values, as shown in Figure 3. Discrete intervals on the *x*-axis represent height ranges, and the vertical extent of each interval represents the number of voxels with a height value within that range. We define a common histogram [39] as follows:

(1) $$h\left(l_{k}\right)=n_{k}$$

where *l_k_* is the observation value and *n_k_* is the total number of data with observation *l_k_*. If the *y* coordinate of a voxel is equal to *l_k_*, the variable *n_k_* will be increased by 1.

A fraction of ground has a smooth, horizontal surface. The 3D sensor cannot pass through the solid ground surface, and no data is scanned below the ground surface. Hence, the height distribution of this ground fraction is highly localized within (−*h*_1_ − ∆, −*h*_1_ + ∆), as illustrated in Figure 3(a). A non-ground object has a vertical surface on the ground. The height distribution of a non-ground object has an evenly localized distribution within (−*h*_1_ + ∆, *h*_2_), as shown in Figure 3(b), where *h*_2_ is the upper extent of the 3D sensor’s range.

**Figure 4.**
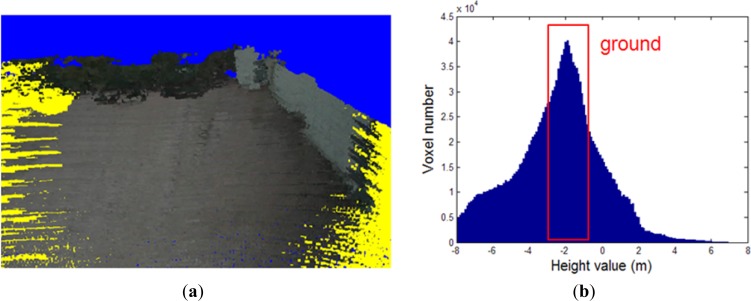
Height histogram generated from voxels in the voxel map. (**a**) The voxel map. (**b**) The height histogram of the voxel map in (a).

We create a height histogram as shown in Figure 4(b), from the voxels in the voxel map as shown in Figure 4(a). We estimate the 3D height value *h*_1_ as that whose voxel count number is the peak of the histogram. The voxels contributing to the interval (−*h*_1_ − ∆, −*h*_1_ + ∆) correspond to the ground surface.

**Figure 5.**
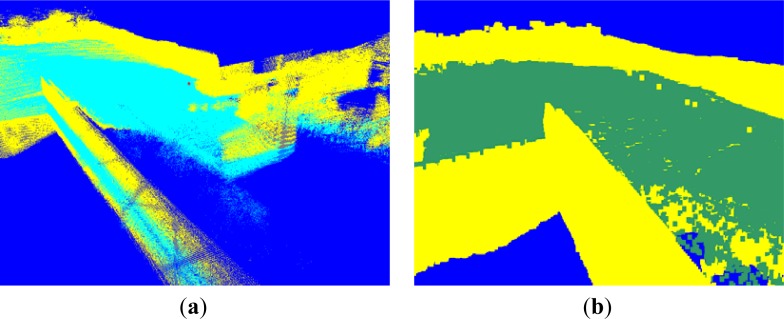
Ground segmentation in the voxel map. (**a**) Rough ground segmentation of the voxel map based on the height histogram. (**b**) Ground segmentation in the voxel map using the height histogram method with the proposed GMRF model.

By applying this estimated height value as a threshold for ground segmentation, we obtain the result shown in Figure 5(a), where the voxels in cyan and yellow represent the ground and non-ground data, respectively. We can see that some regions below the ground are recognized as ground data. This rough segmentation method does not generate all ground data, because the configuration is only determined using a local height.

### 3.2. Refining Process for Ground Segmentation

When we segment ground data using the threshold generated from the 3D histogram method, some errors exist in the segmentation result. In order to remove them in the rough ground segmentation result, we explain a technique that determines the data configuration based on local and neighboring observations in the generated voxel map. We append the GMRF model definition at the end of this paper to explain our method’s theoretical background.

When we apply the GMRF to ground segmentation in a 3D voxel map, we first determine a set of voxels whose configurations imply a high probability of being in the ground class. If the *y* coordinate of a 3D voxel is in the range −*h*_1_ − ∆ to −*h*_1_ + ∆, then the configuration of this voxel is toward the ground class. This step represents a rough ground segmentation process that produces dataset *G*_1_. The voxels located outside this range are grouped into dataset *G*_2_, whose configurations are toward the non-ground class. We use this method to estimate probabilities for each configuration of voxels in the voxel map.

As mentioned in Section 3.1, *G*_1_ contains some non-ground data and *G*_2_ does not contain all non-ground data, because the configuration is determined using only a local height value. Next, we apply the GMRF model designed in Appendix to classify the configurations of voxels into ground or non-ground classes.

The voxels with a ground configuration are grouped into dataset *G*_1_′, whose configurations are determined as the ground class. The voxels in *G*_1_′ are represented as the green region in Figure 5(b). Regions containing non-ground voxels are grouped into dataset *G*_2_′. If a voxel *s* ∈ *G*_1_′ maps onto a pixel in a 2D image, we determine this pixel to be a ground pixel. If not, this pixel is a non-ground pixel.

## 4. 3D Boundary Estimation for Non-Ground Objects

When mobile robots detect information about the surrounding terrain, some parts of objects are outside the range of measurement of their 3D sensors. For example, in Figure 1(c), we can see that the top of the building is missing in the terrain reconstruction result. However, objects such as buildings and vegetation can be seen completely in the 2D image of Figure 1(b), captured by the mobile robot’s camera. In this section, we explain a 3D boundary estimation method for non-ground objects. This solves the problem of recovering unsensed regions by estimating the top boundary of an object. Our proposed boundary estimation process consists of two steps. First, we find the boundary between the object and the background in a 2D image. Next, we find the boundary’s 3D coordinates using an inverse projection from 3D points to 2D pixels.

### 4.1. Boundary Detection of Foreground Objects in 2D Images

Mobile robots require real-time boundary detection. Hence, we apply a simple kernel-based boundary detection method to estimate image gradients and detect the foreground and background in a 2D image. To account for noise in the image, we use dilation and erosion methods to smooth the boundary detection result.

**Figure 6.**
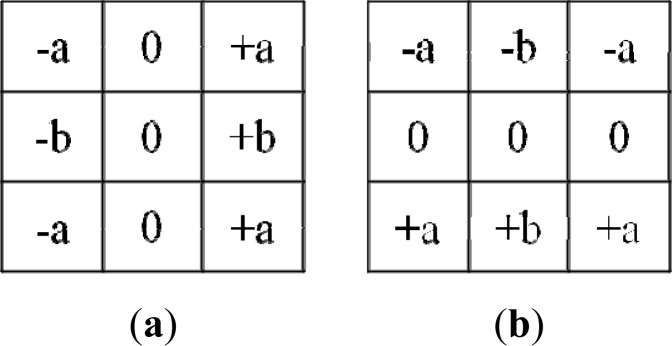
Kernel matrices for boundary detection. (**a**) Horizontal kernel. (**b**) Vertical kernel.

We define a horizontal kernel to detect the boundary in the horizontal direction, and a vertical kernel to detect the boundary in the vertical direction, as shown in Figure 6.

By computing the convolutions *L_x_*(*x*, *y*) and *L_y_*(*x*, *y*) with the kernels in Figure 6, the horizontal and vertical changes in a pixel (*x*, *y*) are formulated as follows:

(2) $$L_{x}\left(x,y\right)=-\mathrm{ap}\left(x-1,y+1\right)+\mathrm{ap}\left(x+1,y+1\right)-\mathrm{bp}\left(x-1,y\right)+\mathrm{bp}\left(x+1,y\right)-\mathrm{ap}\left(x-1,y-1\right)+\mathrm{ap}\left(x+1,y-1\right)$$

(3) $$L_{y}\left(x,y\right)=\mathrm{ap}\left(x-1,\;y-1\right)-\mathrm{ap}\left(x-1,\;y+1\right)+\mathrm{bp}\left(x,y-1\right)-\mathrm{bp}\left(x,y+1\right)+\mathrm{ap}\left(x+1,y-1\right)-\mathrm{ap}\left(x+1,y+1\right)$$

where *a* and *b* are non-zero constants. The gradient of the change in the pixel (*x, y*) is formulated as follows:

(4) $$\nabla L=\sqrt{k_{x}L_{x}{\left(x,y\right)}^{2}+k_{y}L_{y}{\left(x,y\right)}^{2}}$$

The coefficients *k_x_* and *k_y_* affect the weight value of the horizontal and vertical changes, respectively. In this project, we detect the boundary between foreground objects and background data, such as ground pixels and sky pixels. Foreground objects are always located below or above the background in a 2D image. The vertical changes affect the boundary more than the horizontal changes. Therefore, *k_y_* is larger than *k_x_* for all scenarios in our project.

If the change in pixel (*x*, *y*) is large, we consider that this pixel is likely to be on the boundary. Thus, if the magnitude ∇*L*(*x*, *y*) is larger than some threshold, we determine the pixel (*x*, *y*) to be a boundary pixel, at least temporarily.

**Figure 7.**
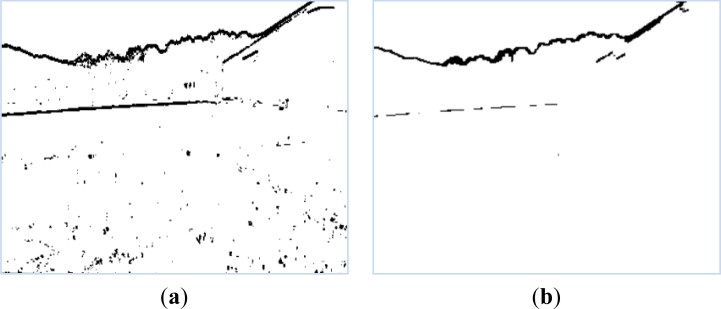
Foreground objects boundary detection. (**a**) Boundary detection result using kernel-based method. (**b**) Removing noise from the boundary detection result using the dilation and erosion methods.

Figure 7(a) shows a binary image of the boundary detection result for Figure 1(b). We define *p*(*x*, *y*) = 0 for the black pixels to represent boundary data, and *p*(*x*, *y*) = 1 for the white pixels to represent non-boundary data.

**Figure 8.**
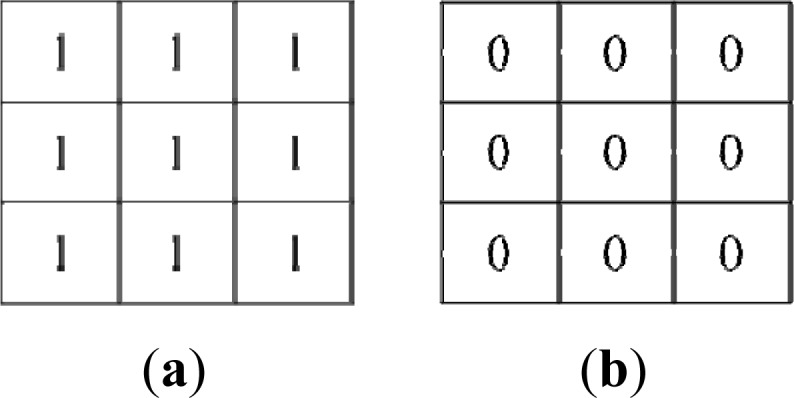
Erosion and dilation masks. (**a**) The erosion mask *B*_1_. (**b**) The dilation mask *B*_2_.

We find that some noise exists in the boundary detection result. To remove this, we apply dilation and erosion filters. We firstly apply erosion process to remove the noise in the boundary detection result. The erosion process is performed by extending the background region in 'white' using an erosion mask, as shown in Figure 8(a).

We shift the erosion mask across the image and generate a boundary detection result *E* by the convolution function on image *A* with the erosion mask *B*_1_, formulated as follows:

(5) $$E\left(x,y\right)=\{\begin{array}{ll} 1 & \mathrm{if}\;\sum_{-1\leq i\leq 1}\sum_{-1\leq j\leq 1}A\left(x+i,y+j\right)⊕B_{1}\left(i,j\right)=9\\ 0 & \mathrm{if}\;\sum_{-1\leq i\leq 1}\sum_{-1\leq j\leq 1}A\left(x+i,y+j\right)⊕B_{1}\left(i,j\right)\neq 9 \end{array}$$

where the symbol ⊕ stands for the operation “exclusive or”.

Using the erosion process to remove the noise, we find that some boundary pixels are filtered out. Subsequently, we apply the dilation process to recover the filtered boundary pixels. The dilation process is performed by extending the boundary region in “black” using a dilation mask, as shown in Figure 8(b).

We shift the dilation mask across the image and generate a boundary detection result D by the convolution function on image *E* with the dilation mask *B*_2_, formulated as follows:

(6) $$D\left(x,y\right)=\{\begin{array}{cc} 1 & \mathrm{if}\;\sum_{-1\leq i\leq 1}\sum_{-1\leq j\leq 1}A\left(x+i,y+j\right)⊕B_{2}\left(i,j\right)=0\\ A\left(x,y\right) & \mathrm{if}\;\sum_{-1\leq i\leq 1}\sum_{-1\leq j\leq 1}A\left(x+i,y+j\right)⊕B_{2}\left(i,j\right)\neq 0 \end{array}$$

The experimental result of removing noise from Figure 7(a) is shown in Figure 7(b). The boundary between the foreground and background is extracted as the top black curve in Figure 7(b).

### 4.2. 3D Boundary Estimation in 3D Textured Terrain Mesh

In this section, we propose a 3D boundary estimation method for the 3D terrain mesh that allows us to recover the complete shape of non-ground objects. Using the boundary detection between the foreground object and the background in a 2D image, we find the boundary’s 3D coordinates by projecting from 2D pixels to 3D vertices secondly.

From the ground data segmentation results, we consider a 3D non-ground voxel in the terrain mesh as part of the foreground object. This is because background data, such as sky, cannot be sensed.

**Figure 9.**
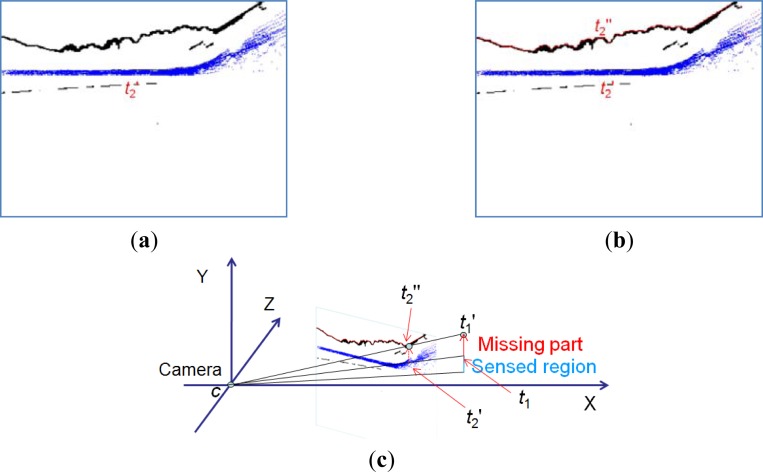
Boundary detection for non-ground objects. (**a**) Projection results from vertices in dataset *T*_1_. (**b**) Non-ground objects boundary detection results in 2D image. (**c**) 3D boundary detection process.

When non-ground voxels in *G*_2_′ are inserted into the terrain mesh, the updated vertices are categorized into a non-ground vertex dataset *T*_1_. By projecting from *t*_1_(*x*_1_, *y*_1_, *z*_1_), *t*_1_∈*T*_1_, to the 2D image, we map *t*_1_ to the pixel *t*_2_′ in the 2D image given by the boundary detection result. These *t*_2_′ make up the dataset *T*_2_, which is shown as the blue pixels in Figure 9(a).

We search for a boundary pixel *t*_2_″ above *t*_2_′ as the object’s top pixel, as indicated in red in Figure 9(b). From the true top location *t*_2_″ in the 2D image, we estimate the height value for each object vertex.

We find the *y* coordinates of the boundary using an inverse projection from 2D pixels to 3D points, as shown in Figure 9(c). We place the center of the camera at the origin. The projection ray from the origin to the non-ground object vertex gives an estimate of the height of that vertex.

The direction of the vector $\vec{{{\mathrm{Ct}}_{1}}^{\prime }}$ is the same as that of $\vec{{{\mathrm{Ct}}_{2}}^{\prime \prime }}$, from the camera to the estimated 3D point *t*_1_(*x*_1_, *y*_1_, *z*_1_). After the camera transforms by the rotation matrix *R*, the vector $\vec{{{\mathrm{Ct}}_{2}}^{\prime \prime }}$ is derived as $\vec{{{\mathrm{Ct}}_{2}}^{\prime \prime }}=R\left(\vec{\mathrm{Co}}+\vec{{{\mathrm{ot}}_{2}}^{\prime \prime }}\right)$. Therefore, we formulate that:

(7) $$\vec{{{\mathrm{Ct}}_{1}}^{\prime }}=\lambda \vec{{{\mathrm{Ct}}_{2}}^{\prime \prime }}=\lambda R(\vec{\mathrm{Co}}+\vec{{{\mathrm{ot}}_{2}}^{\prime \prime }})$$

In Equation (7), *λ* is a scalar number; the vector from the camera to the principal point is $\vec{\mathrm{Co}}=\left[{ɛ}_{x},{ɛ}_{y},f\right]$; the vector from the principal point to the estimated vertex of boundary is $\vec{{{\mathrm{ot}}_{2}}^{\prime \prime }}$. We define a vector [*x*″, *y*″, *z*″] as the result of $R\left(\vec{\mathrm{Co}}+\vec{{{\mathrm{ot}}_{2}}^{\prime \prime }}\right)$. According to matrix equivalence, the Equation (7) is derived as:

(8) $$\bar{{{\mathrm{Ct}}_{1}}^{\prime }}=\left[x_{1},{y_{1}}^{\prime },z_{1}\right]=\left[\lambda x\prime \prime ,\lambda y\prime \prime \lambda z\prime \prime \right]∼\left[x\prime \prime z_{1}/z\prime \prime ,y\prime \prime z_{1}/z\prime \prime ,z_{1}\right]$$

We derive the estimated height value as: *y*_1_′ = *y*″*z*_1_/*z*″ or *y*_1_′ = *y*″*x*_1_/*x*″. Then, we reset the height value of the foreground vertex *t*_1_ with (*x*_1_, *y*_1_′, *z*_1_). Because the horizon coordinates (*x*_1_, *z*_1_) of the 3D object vertex are fixed in the terrain mesh, we update the elevation value *y*_1_′ of each object vertex in the terrain mesh to obtain the results shown in Figure 14.

## 5. Experiments and Analysis

In this section, we describe several experiments to analyze the performance of the proposed non-ground object detection and 3D boundary estimation methods. The experiments have been performed in three steps. Firstly, we have reconstructed a voxel map and textured mesh in the virtual environment by integrating frames of 3D point clouds. Next, we have segmented ground voxels in the voxel map using the height histogram method with a GMRF model. Finally, we have estimated object boundaries in the 2D images using the object vertices in the terrain mesh and evaluated the height of each object cell.

Experiments were carried out using a mobile robot with integrated sensors, including a GPS, gyroscope, video camera, and 3D sensor. We used an HDL-64E Velodyne sensor, giving approximately 1.333 million laser shots per second, to scan 3D points in the unknown environment. The valid data range is approximately 70 m from the robot. Our algorithms are implemented on a laptop PC with a 2.82 GHz Intel^®^ Core™2 Quad CPU, a GeForce GTX 275 graphics card, and 4 GB RAM. We drive the robot around an outdoor area of 100 × 100 m^2^, including buildings and trees. The upper parts of these objects are outside the range of the robot’s sensors, but are captured in the 2D images. We also utilized an HDL-32E Velodyne sensor in other two environments, as shown in Figure 15, to investigate the performance of the proposed algorithms.

### 5.1. Performance of the Ground Segmentation Method

In this section, we analyze the ground segmentation results, discuss the accuracy of the model for different densities of terrain map, and show that the proposed algorithm is fast enough to be used in a real-time approach. We apply the proposed height histogram method to estimate the height range of the ground surface, and then use the GMRF model to segment the ground data with the results of the height histogram.

**Figure 10.**
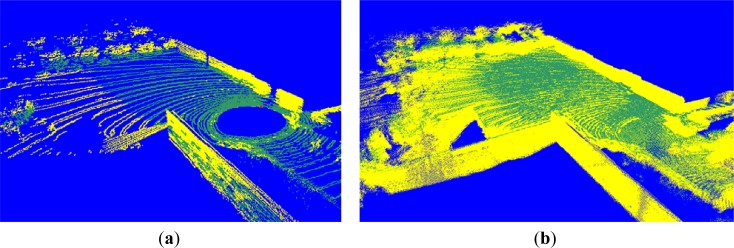
Segmentation results. (**a**) Segmentation result from 88,536 voxels. (**b**) Segmentation result from 1,817,035 voxels.

The voxel map integrated from a few frames has a low density and small point quantity, so that rare neighboring voxels exist centered by a voxel. It is thus difficult to estimate the configurations of terrain voxels. In our projection, we collected 235,940 lasers in a frame, and implemented the ground segmentation once per frame. Figure 10(a) shows the ground segmentation result for the voxel map generated from one frame, which register 88,536 voxels in the terrain model buffer. The ground segmentation took 0.03 s.

We see from Figure 10(a) that the accuracy of the ground segmentation is not high. When we generate a cohesive terrain map integrated from many frames of 3D point clouds, the density is high and the quantity of points is large. Figure 10(b) shows a voxel map made of 1,817,035 voxels, generated from 100 frames. The computation for the ground segmentation result in Figure 10(b) took 0.496 s.

**Figure 11.**
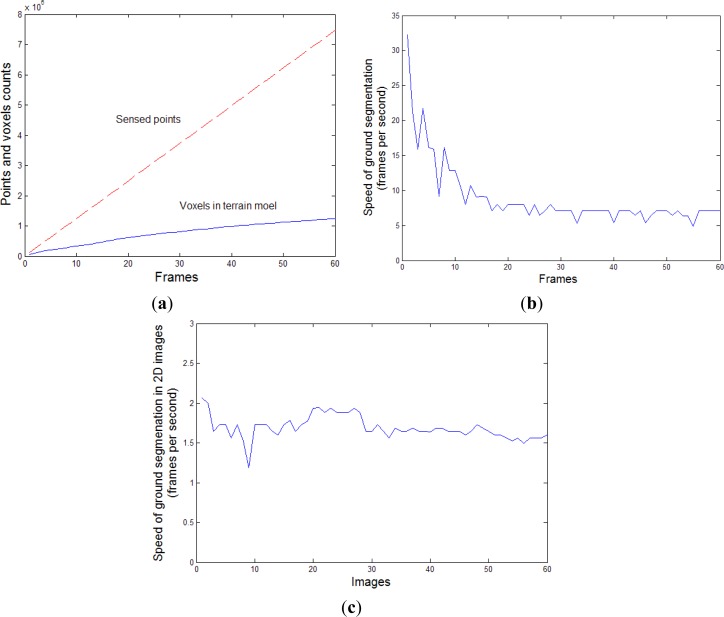
Ground segmentation performance over frames 1∼60. (**a**) Number of sensed points and processed voxels. (**b**) Speed of ground segmentation for the voxel map. (**c**) Speed of ground segmentation for the captured 2D images.

Figure 11(a) shows the numbers of the sensed points and the voxels processed for ground segmentation in frames 1∼60. Figure 11(b) shows the speed of the ground segmentation processing. At the beginning of the testing, only 1.8 × 10^5^ voxels are sensed in the first five frames. The ground segmentation for the voxel map of low density performs a high speed of the ground segmentation, more than 15 fps. As more frames are collected for the voxel map, a higher number of neighboring voxels are included in the computation of the voxel’s configuration, which cause the duration of the ground segmentation processing to increase. When the robot moves faster than 0.4 m/s, there are 1.237 × 10^5^∼1.253 × 10^5^ voxels registered in the voxel map for each frame approximately. The new registered voxels cause a higher computation for GMRF model and the ground segmentation performs a low speed at 6.25 fps averagely. Because the numbers of the new registered voxels are different for each frame, some variances exist in the Figure 11(b). The sensing duration of a frame was 0.177 s. To realize real-time requirement, the ground segmentation duration need to be less than 0.177 s. From the simulation result of Figure 11(b), we can see that the ground segmentation takes less than 0.16 second, which satisfies the real-time requirement. By applying multi-thread programming, we collect the voxel map and implement the ground segmentation in parallel, in order to realize real-time terrain modeling.

We also implemented the GMRF-based ground segmentation method in 2D images. Firstly, we segmented the ground vertices in the terrain mesh and mapped them onto 2D pixels. Next, we segmented all the ground pixels using the GMRF model with the flood-fill algorithm, as proposed by Song [32]. The performance of this approach is shown in Figure 11(c). The duration of ground segmentation in the 2D images with a solution of 320 × 240 pixels is around 0.53 second. Thus, our proposed ground segmentation method in the voxel map is faster than that in 2D images.

### 5.2. Performance of the 3D Boundary Estimation Method

**Figure 12.**
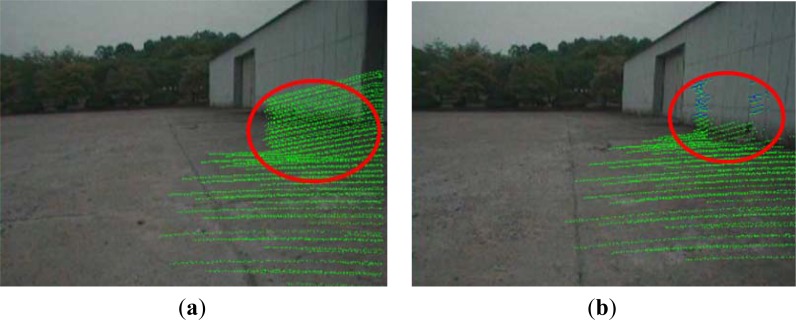
Projection from 3D points of 0.1 frame to a 2D image. (**a**) Projection without calibration processing. (**b**) Projection with calibration processing.

Before terrain reconstruction, we apply a calibration of camera and the Velodyne sensor to the camera. In order to realize real-time terrain reconstruction, we implement the calibration method only once before the terrain reconstruction. In Figure 12(a), the green pixels are projected from the sensed 3D points of 0.1 frame to the captured 2D image, without calibration. We see that the projected pixels do not match their actual position in the 2D image. After the calibration of the projection matrix, the projection results are shown as green pixels in Figure 12(b). We see that the boundary pixels between the building and ground surface match their positions in the 2D image.

**Figure 13.**
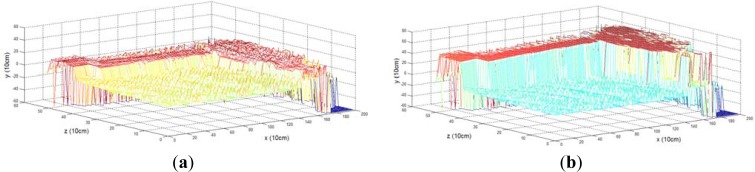
3D boundary estimation results for non-ground objects. (**a**) The height map generated before 3D boundary estimation. (**b**) The height map generated after 3D boundary estimation.

**Figure 14.**
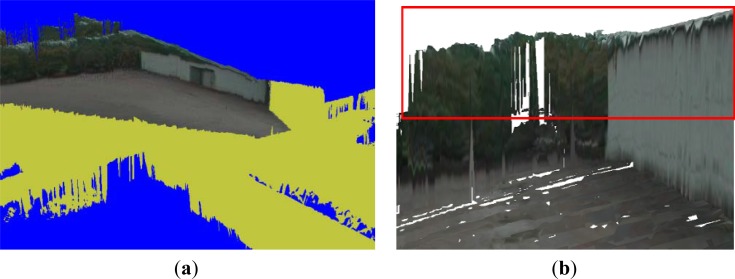
Reconstruction result after 3D boundary estimation for non-ground objects. (**a**) Top view of the terrain reconstruction using the 3D boundary estimation algorithm. (**b**) Terrain refining result when the robot moves forward.

In this section, we investigate the performance of the proposed boundary estimation method by comparing the obtained values with the actual object heights (2.90 m on average). Figure 13(a) shows the height map of the incomplete terrain mesh in Figure 1(c), where the horizon coordinates of vertices correspond to the x-axis and y-axis. Previous interpolation algorithms average the empty region using the surrounding 3D points. However, using our proposed 3D boundary estimation method, we recovered the unsensed parts of foreground objects, which are sensed in the incomplete terrain mesh of Figure 1(c). Figure 13(b) shows a height map generated after 3D boundary estimation, where the estimated height values were close to the actual value. When the non-ground vertex is far from the camera, the slight errors of the boundary detection in 2D image, even a pixel offset, cause erroneous results in 3D boundary detection due to the inverse projection function, expressed as Equation (8). In the simulation result of Figure 13(b), the errors exist in the far-field regions, more than 100 meters to the camera. The variance of the errors was less than 0.8 meter. However, when the robot moves forward, the shape of the recovered parts of unsensed objects is refined as Figure 14(b). The variance of the errors is reduced to 0.31 meter.

The textured terrain mesh with complete objects provides the remote operator with an intuitive 3D scene with foreground objects on the ground. This gives a better representation of the surrounding terrain than that generated directly from the sensed datasets. Whereas Huber’s work [8] rendered far-field regions of 30 m away using a texture billboard, the proposed terrain reconstruction method provides a complete picture of the terrain model for up to 100 m. The rendering speed is more than 25 fps.

By mapping the texture from the 2D image to the terrain mesh, we reconstructed a complete terrain model captured from the quarter view, as shown in Figure 14(a). The reconstructed terrain model contains 576,247 vertices and 477,315 triangles. Because we amount the camera in front of the robot, the vertices in front of the camera are projected to the 2D image so that the front parts of the terrain model are represented with the projected texture. To denote the vertices that are not projected from the 2D image, we render yellow vertices in the terrain visualization result. As we see from Figure 1(c), the upper parts of buildings and trees are not sensed. Using the 3D boundary estimation method, we recovered these missing parts from the image of Figure 1(a). After the robot moved 40 meters forward, the terrain reconstruction result was refined as Figure 14(b).

**Figure 15.**
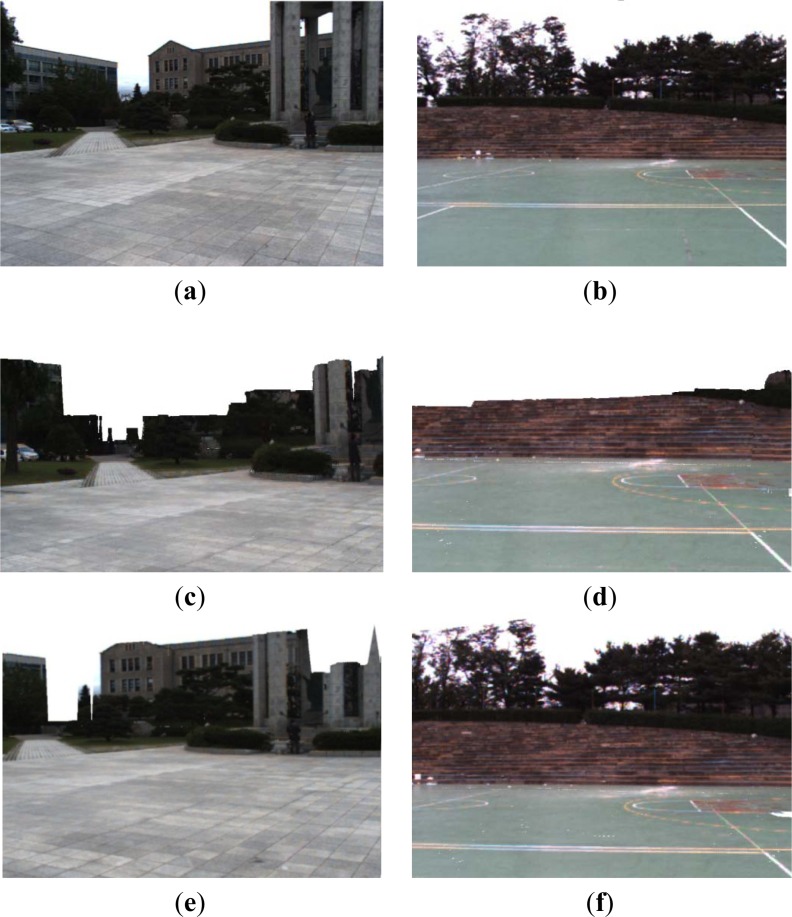
Other simulation results using the proposed terrain reconstruction methods. (**a**,**b**) Captured 2D images. (**c**,**d**) Textured terrain meshes generated from sensed point clouds directly. (**e**,**f**) Terrain reconstruction using the proposed 3D boundary detection from (c) and (d) respectively.

Figure 15 shows some other simulation results for the proposed terrain reconstruction results. The images of Figure 15(a,b) shows the 2D images captured in front of the mobile robot. The textured meshes in Figure 15(c,d) shows the terrain reconstruction results, where the upper parts of buildings and trees cannot be sensed. The textured meshes in Figure 15(e,f) shows the complete scene recovery result from the terrain mesh in Figure 15(c,d) respectively, using the 3D boundary estimation method. Because there was no sensed vertices of the non-ground objects in far-field, the 3D boundary estimation algorithm were not implemented for these objects. Because the camera does not capture the top of the figure object in the image of Figure 15(a), the 2D boundary pixels of the figure object are not detected. The reconstructed terrain mesh of Figure 15(e) covered 73.4% of the boundary in 2D image of Figure 15(a), and the mesh of Figure 15(f) covered 91.3% of the image of Figure 15(b). We view the realistic objects in 3D terrain mesh effectively with 3D complete scene for foreground objects on the ground.

## 6. Conclusions

In this paper, we described a ground segmentation technique and a non-ground boundary estimation method for automated surveying and mapping by mobile robots. The methods are shown to be effective in an outdoor environment for a mobile robot with a 3D sensor, video camera, GPS, and gyroscope. The datasets from multiple sensors are integrated in the forms of voxel map and textured mesh in order to develop a terrain modeling system.

During remote operation, it is not convenient to classify non-ground objects using 3D point clouds. Ground segmentation is required for the classification of ground surface and non-ground objects, but traditional methods for 2D images must be implemented for each captured image, leading to a huge computational cost. To overcome this problem, we developed a ground segmentation approach using a height histogram and GMRF model in the reconstructed terrain voxel map. We showed that our method is faster than segmentation algorithms based on image processing.

To represent non-ground objects outside the measurement range of the robot’s 3D sensors, conventional interpolation algorithms are applied. However, it is difficult for these methods to recover the shape of large objects. To solve this problem, we described a 3D boundary estimation method that estimates the true height value of an object from its boundary in the 2D image. The actual height of objects is estimated using a projection from 2D pixels to 3D vertices. This method enables real-time terrain modeling and provides the remote robot operator with photorealistic visualization support.

We tested our approach using a mobile robot mounted with integrated sensors. The simulation results demonstrate the intuitive visualization performance of the proposed method in a large-scale environment. The speed of terrain modeling and photorealistic visualization satisfies the constraints of real-time operation. Our works are compatible with global information database collection, streetscape representation, augmented reality and other multimedia applications.

However, in the ground segmentation results, if the computed voxel is far from the robot, it is difficult to evaluate the accurate probability for the configurations in GMRF model. Even in the rough terrain, such as vegetation areas, the irregular object shape distribution will cause the errors in ground segmentation results. We implement the 3D boundary estimation algorithm from textured mesh, which cannot model the empty areas inside the objects, such as trees. We need to improve and optimize the algorithms to deal with these problems in future.

## Appendix GMRF Model Definition

The configuration of a voxel also depends on its connected neighbors. This phenomenon follows the property of GMRF. Hence, we apply GMRF to segment the ground data from the segmentation result computed by the height histogram method.

We define *S* as a set of voxel sites. Any *s*∈*S* is a voxel location (*x_s_*, *y_s_*, *z_s_*) in the voxel map. The random vector *X* = {*X_S_*} on *S* has a value *O*. In our application, *O* represents a vector consisting of a height observation variable and a configuration variable. The configuration variable has a ground value and a non-ground value.

A neighborhood system for *s* contains all sites within a distance *r* (*r* ≥ 0) from *s*, defined as *N* = {*N_s_*| *s*∈*S*}, where *N_s_* is the neighbor set for site *s* given by:

(9) $$N_{s}=\{s^{\prime }\in S\|x_{s}-x_{s^{\prime }}|+|y_{s}-y_{s^{\prime }}|+|z_{s}-Z_{s^{\prime }}|\leq r,s\neq s^{\prime }\}$$

We define a clique as a set neighboring a given site. In our application, a clique contains the given voxel and its neighboring voxels within a distance of *r* = 1. A clique set *C* is defined as a collection of single-site *C*_1_ and pair-site *C*_2_ cliques. *C* satisfies the condition that each pair of distinct sites in *C* is a neighbor, defined as follows:

(10) $$C=C_{1}\cup C_{2}$$

(11) $$C_{1}=\left\{s\right\}\;\text{for}\;s\in S$$

(12) $$C_{2}=\left\{\left\{s,s^{\prime }\right\}|s^{\prime }\in N_{i},s\in S\right\}$$

Based on its MRF property, the configuration at site *s* only depends on the configuration of its neighboring sites. We find the best possible configuration *f** for site *s* using the following optimum solution:

(13) $$f*\left(s\right)=\text{arg}\underset{f}{\text{max}}p\left(X_{s}=f|X_{t}=d,\forall t\in S\right)$$

To evaluate the PDF in Equation (11), we apply the Gibbs distribution [10], following the Hammersley-Clifford theorem. The probability of a site’s configuration is calculated as:

(14) $$p\left(f\right)=Z^{-1}e^{-\frac{1}{T}U\left(f\right)}$$

(15) $$U\left(f\right)=\sum_{c\in C}V_{c}\left(f\right)$$

(16) $$Z=\sum_{f}e^{-\frac{1}{T}U\left(f\right)}$$

The potential function *V_c_*(*f*) evaluates the effect of neighbor sites in clique *c*∈*C*, and the energy function *U*(*f*) in Equation (14) is defined as the sum of the impacts of clique set *C*. The probability should satisfy the condition 0 ≤ *p*(*f*) ≤ 1. The partition function *Z* is defined in Equation (16). To normalize *p*(*f*), we divide the sum of the exponential functions derived from all possible configurations by the partition function *Z*. The constant *T* is referred to as the temperature factor in Gibbs’ theory, and it controls the deviation of the distribution of *p*(*f*) in MRF.

According to Bayes’ rule, the solution of Equation (13) is as follows:

(17) $$f*\left(s\right)=\text{arg}\underset{f}{\text{max}}p\left(f|d\right)=\text{arg}\underset{f}{\text{max}}p\left(d|f\right)p\left(f\right)=\text{arg}\underset{f}{\text{min}}U\left(f|d\right)=\text{arg}\underset{f}{\text{min}}\left\{U\left(d|f\right)+U\left(f\right)\right\}$$

The energy function *U*(*d*|*f*) + *U*(*f*) evaluates the effect of neighbor sites in single- and pair-site potential cliques as follows:

(18) $$U\left(d|f\right)+U\left(f\right)=\sum_{s\in C_{1}}V_{1}\left(f_{s}\right)+\sum_{\left\{s,s^{\prime }\right\}\in C_{2}}V_{2}\left(f_{s},f_{s^{\prime }}\right)+\sum_{s\in C_{1}}V_{1}\left(d_{s}|f_{s}\right)+\sum_{\left\{s,s^{\prime }\right\}\in C_{2}}V_{2}\left(d_{s},d_{s^{\prime }}|f_{s},f_{s^{\prime }}\right)$$

When we apply the GMRF in the voxel map, we define a voxel site *v*∈*S*. The observation *h_v_* is the height value of voxel *v*. Evaluation of the clique potential functions *V*_1_(*f_v_*) and *V*_1_(*h_v_*|*f_v_*) depends on the local configuration and observations of clique *C*_1_. The clique potential functions *V*_2_(*f_v_*, *f_v’_*) and *V*_2_(*h_v_*, *h_v’_*|*f_v_*, *f_v’_*) evaluate the pair-site consistency of clique *C*_2_. The clique potential functions are formulated as follows:

(19) $$V_{1}\left(f_{v}\right)=\{\begin{array}{lllllll} -\alpha & \mathrm{if}(v\in G_{1} & \mathrm{and} & f_{v}=\mathrm{ground}) & \mathrm{or}(v\in G_{2} & \mathrm{and} & f_{v}=\mathrm{nonground})\\ +\alpha & \mathrm{if}(v\in G_{2} & \mathrm{and} & f_{v}=\mathrm{ground}) & \mathrm{or}(v\in G_{1} & \mathrm{and} & f_{v}=\mathrm{nonground}) \end{array}$$

(20) $$V_{1}\left(h_{v}|f_{v}\right)=\{\begin{array}{ll} -\alpha & \mathrm{if}\left(h_{v}\in R\left(f_{v}\right)\right)\\ +\alpha & \mathrm{if}\left(h_{v}\notin R\left(f_{v}\right)\right) \end{array}$$

(21) $$V_{2}\left(f_{v},f_{v^{\prime }}\right)=\{\begin{array}{ll} -\beta & \mathrm{if}\left(f_{v}=f_{v}\right)\\ +\beta & \mathrm{if}\left(f_{v}\neq f_{v}\right) \end{array}$$

(22) $$V_{2}\left(h_{v},h_{v^{\prime }}|f_{v},f_{v^{\prime }}\right)=\{\begin{array}{ll} -\gamma e^{-\left\|h_{v}-h_{v^{\prime }}\right\|} & \mathrm{if}\left(f_{v}=f_{v^{\prime }}\right)\\ +\gamma e^{-\left\|h_{v}-h_{v^{\prime }}\right\|} & \mathrm{if}\left(f_{v}\neq f_{v^{\prime }}\right) \end{array}$$

The constants *α*, *β*, and *γ* are positive values. The configuration *f_v_* depends on whether voxel *v* belongs to the ground dataset or the non-ground dataset. The function *R*(*f_v_*) returns the height range of voxels with the configuration *f_v_*, and the expression ||*h_v_* – *h_v’_*|| gives the height difference between observations *h_v_* and *h_v’_*. We solve Equation (17) using the potential functions defined in Equations (18)–(22), and label the configuration of each voxel.
